# Predictors of Concomitant Pulmonary Involvement in Hepatic Cystic Echinococcosis: A Clinical Risk Stratification Model

**DOI:** 10.1007/s11686-026-01291-4

**Published:** 2026-05-02

**Authors:** Oğuzhan Taş, Mehmet Ali Eryazğan

**Affiliations:** 1 Department of General Surgery, Faculty of Medicine, Aksaray Training and Research Hospital, Aksaray University, Aksaray, Turkey; 2 Department of Thoracic Surgery, Faculty of Medicine, Aksaray Training and Research Hospital, Aksaray University, Aksaray, Turkey

**Keywords:** Cystic echinococcosis, Hepatopulmonary hydatidosis, Segmental liver anatomy,, WHO cyst classification, Risk stratification

## Abstract

**Purpose:**

Hepatopulmonary hydatidosis (HPH) is a clinically relevant presentation of hepatic cystic echinococcosis (CE) in which pulmonary involvement is present at the time of diagnosis. Reliable identification of patients at risk remains challenging, and indiscriminate thoracic imaging may lead to unnecessary investigations. This study aimed to identify hepatic predictors associated with concomitant pulmonary involvement and to develop a simple risk stratification model to support selective thoracic imaging.

**Methods:**

We conducted a retrospective cohort study of patients with confirmed hepatic CE followed at a single tertiary center. Cyst activity was classified according to the World Health Organization (WHO) staging system, and anatomical distribution was assessed using a segment-based classification. Multivariable logistic regression was performed to identify predictors of HPH. A point-based clinical risk score (HepatoMAP) was derived by combining cyst activity and anatomical distribution. Model discrimination and calibration were assessed using receiver operating characteristic (ROC) analysis, bootstrap validation, and calibration plots.

**Results:**

Among 292 patients, 23 (7.8%) had hepatopulmonary hydatidosis (HPH) at initial diagnosis. Active cysts (WHO CE1–2) were strongly associated with HPH (91.3% in HPH vs. 33.2% in hepatic-only disease, *p* < 0.001) and remained the only independent predictor in multivariable analysis. The HepatoMAP score demonstrated good discrimination (AUC 0.83) with good calibration (bootstrap-corrected slope 0.97). No cases of HPH were observed in patients with low-risk scores (0–1 points), whereas HPH occurred predominantly in patients with scores ≥ 3.

**Conclusion:**

In hepatic CE, concomitant pulmonary involvement at baseline was strongly associated with cyst activity and showed a structured but non-independent relationship with segmental topography. The HepatoMAP score showed promising rule-out characteristics in this cohort and may support more selective use of thoracic imaging. Prospective external validation is required before routine clinical implementation.

**Supplementary Information:**

The online version contains supplementary material available at 10.1007/s11686-026-01291-4.

## Introduction

Cystic echinococcosis (CE) is a zoonotic parasitic disease caused by the larval stage of *Echinococcus granulosus* and remains a significant public health problem in endemic regions worldwide [1–3]. The liver and lungs represent the two most commonly involved organs, reflecting both portal venous filtration and systemic dissemination pathways [4]. Although hepatic involvement predominates, pulmonary disease may occur either concurrently or secondarily, leading to hepatopulmonary hydatidosis (HPH), a clinically relevant manifestation associated with increased diagnostic complexity, therapeutic challenges, and prolonged follow-up [5–7]. Reported frequencies of pulmonary involvement among patients with hepatic CE vary widely, largely owing to heterogeneity in study populations, imaging strategies, and duration of surveillance [8].

The biological behavior of CE is heterogeneous and influenced by cyst viability, host-related factors, and anatomical conditions. The development of HPH is generally attributed to a combination of hematogenous dissemination and transdiaphragmatic spread, although the relative contribution of these mechanisms remains incompletely defined [9]. Viable cysts may exhibit higher intracystic pressure, increased permeability, and a greater propensity for rupture or systemic spread compared with inactive or degenerative lesions. In routine clinical practice, cyst biological activity is most commonly assessed using the World Health Organization Informal Working Group on Echinococcosis (WHO-IWGE) classification, which stratifies cysts into active (CE1–CE2), transitional (CE3), and inactive (CE4–CE5) stages [2, 10]. However, cyst activity alone does not adequately explain why pulmonary involvement develops in only a subset of patients with hepatic CE, indicating that additional modifiers of dissemination risk are likely involved.

Hepatic anatomical distribution and cyst burden have long been recognized as potential determinants of disease behavior in CE, particularly with respect to complications and extrahepatic spread [4]. Beyond hydatid disease, segmental liver anatomy has been shown to influence disease distribution and clinical outcomes in a variety of hepatic conditions, underscoring the biological plausibility of anatomical modifiers [11, 12]. Segment-based assessments may capture clinically relevant information by reflecting cyst proximity to vascular structures, diaphragmatic surfaces, and hepatic venous outflow, all of which could plausibly influence the likelihood of thoracic involvement. From an infectious disease perspective, anatomical factors may therefore interact with cyst viability to shape dissemination patterns rather than acting as isolated determinants.

In the absence of reliable predictors for HPH, thoracic imaging is often applied liberally in patients with hepatic CE, particularly in endemic settings such as Türkiye, where disease burden remains substantial [13, 14]. Although thoracic computed tomography is highly sensitive for detecting pulmonary hydatid disease, routine imaging of all patients with hepatic CE may result in unnecessary radiation exposure, increased healthcare costs, and incidental findings of uncertain clinical relevance [7, 8]. A pragmatic, clinically applicable risk stratification approach capable of identifying patients at negligible risk for HPH could therefore support more selective imaging strategies without compromising patient safety.

Against this background, we aimed to identify hepatic features associated with hepatopulmonary hydatidosis in patients with hepatic cystic echinococcosis and to develop a simple, clinically applicable risk stratification model. By integrating cyst biological activity with hepatic anatomical distribution, our objective was to explore whether these features could support estimation of baseline HPH risk and to guide the selective use of thoracic imaging in routine clinical practice.

## Materials and Methods

### Study Design and Population

This single-center retrospective study included patients diagnosed with CE between January 2015 and January 2025 at Aksaray Training and Research Hospital, Turkey. A total of 292 consecutive patients diagnosed with cystic echinococcosis (CE) based on clinical and radiologic criteria were identified from institutional radiology and clinical databases. Patients who had previously been diagnosed and treated for hydatid cysts and patients with missing data were excluded. Patients were categorized into four mutually exclusive groups:

1. Hepatic only CE
2. Hepatopulmonary hydatidosis (HPH),
3. Single pulmonary hydatid disease (SPH),
4. Other organ involvement

Because the study sought to identify hepatic predictors associated with concomitant pulmonary involvement, the primary analytic cohort included only patients with hepatic-only CE and HPH (n = 267). Patients with isolated pulmonary or non-hepatic extrahepatic disease were excluded from predictive modeling, as they lack evaluable hepatic cyst characteristics.

### Imaging Assessment

Case identification and baseline pulmonary involvement status were determined retrospectively from institutional radiology reports and clinical records. All available abdominal radiologic studies—including abdominal ultrasonography (USG), computed tomography (CT), and magnetic resonance imaging (MRI)—were subsequently re-reviewed by the authors for hepatic segmental localization and SARAY assignment. Thoracic screening in patients with hepatic CE was based on chest radiography performed as part of routine clinical evaluation. Patients with suspected pulmonary hydatid involvement underwent thoracic CT for further confirmation and characterization. All patients classified as having pulmonary involvement (HPH or SPH) had thoracic CT confirmation. Because of the retrospective design, formal blinding to clinical status was not feasible.

### Hepatic Cyst Classification

Hepatic cysts were staged according to the WHO-IWGE classification (CE1–CE5), which categorizes lesions based on radiologic morphology and viability. For patients with multiple hepatic cysts, the dominant lesion (largest, or most radiologically active when sizes were similar) was used for primary analysis. This dominant-cyst approach was adopted to preserve patient-level modeling and to avoid within-patient clustering or disproportionate weighting of individuals with multiple lesions.

### SARAY Topographic System

SARAY (Segmental Anatomy–Related Y-Scale) is a segment-based topographic framework developed for this study to group hepatic segments according to shared cranial orientation and posterior–superior predominance, relative parenchymal volume, and inferred relationship to the diaphragmatic dome. The rationale was not limited to local diaphragmatic adjacency alone. Rather, the grouping was intended to capture broader anatomical conditions that might influence both cyst establishment and clinically relevant thoracic involvement, including differential surface orientation, regional parenchymal volume, and the possibility that larger posterior–superior right-lobe territories may intercept a greater proportion of portal venous oncospheres before involution. Segment assignment was based on Couinaud anatomy using available USG and CT/MRI reports. For all analyses, each patient’s dominant cyst (largest or most active) was classified into one of three SARAY categories:

- **SARAY 0 – Medial/Central Segments (S1, S4, S5):** These segments occupy a medial or central position, with lower parenchymal volume and less posterior–superior orientation. Their superior surface exposure and diaphragmatic interface are limited.
- **SARAY 1 – Lateral Segments (S2, S3):** These segments occupy a lateral position on the left lobe and display partial or oblique cranial orientation. Their superior surface area is intermediate, providing limited but non-negligible diaphragmatic adjacency.
- **SARAY 2 – Posterior–Superior Right-Lobe Cluster (S6, S7, S8):** This contiguous anatomic region constitutes the largest cranial surface and encompasses the dominant posterior–superior dome. Prior volumetric studies have shown that these segments account for a substantial proportion of right-lobe parenchymal volume and cranial surface exposure, making them anatomically distinct from other hepatic regions. SARAY 2 therefore represents the segmental zone with the broadest superior orientation.

**Fig. 1:**
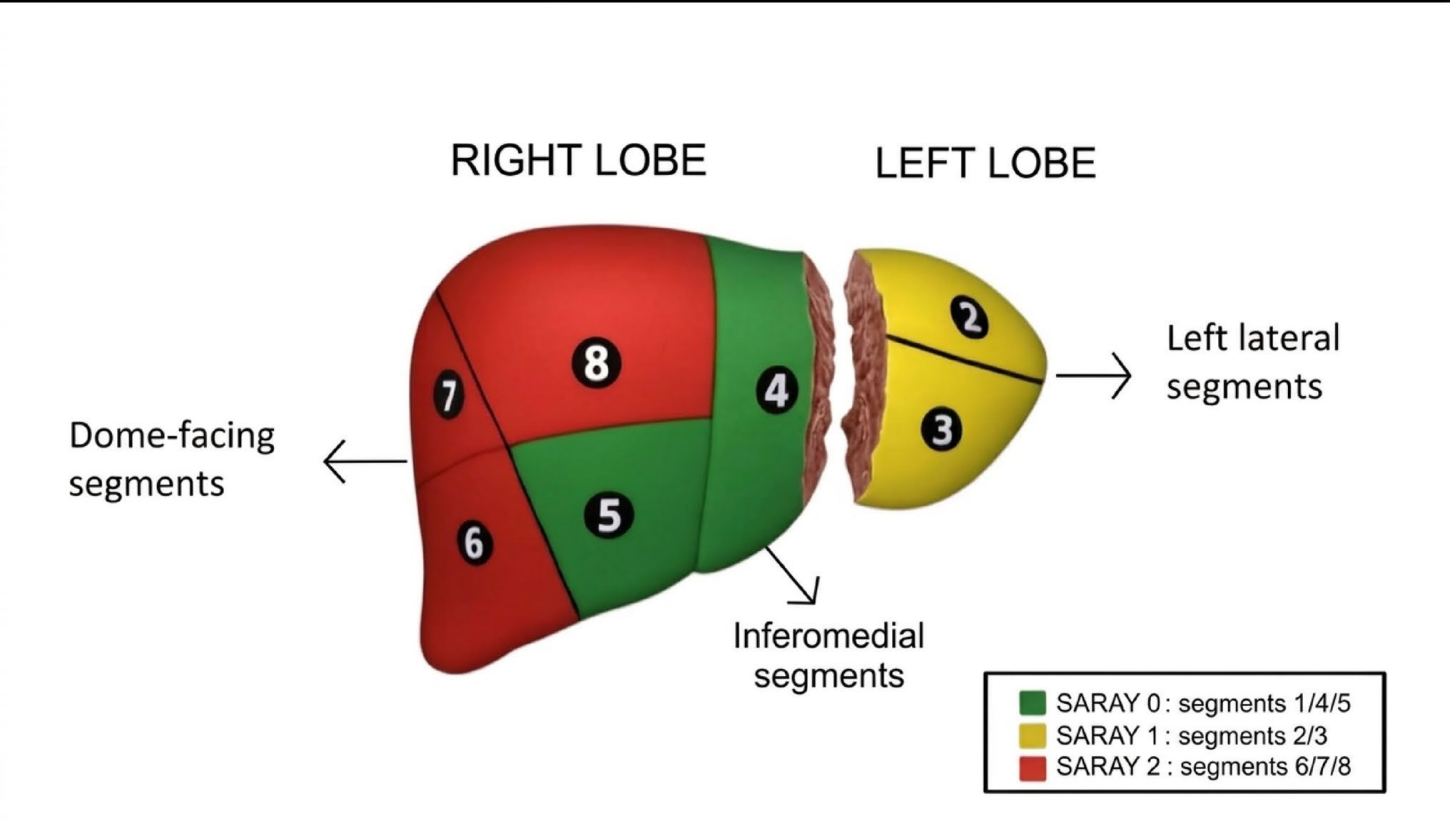
SARAY segmental topographic classification in hepatic cystic echinococcosis. SARAY (Segmental Anatomy–Related Y-Scale) is a segment-based topographic framework that groups hepatic segments according to broad cranial orientation, posterior–superior predominance, relative regional volume, and inferred diaphragmatic relationship. SARAY 0 (green) includes medial/central segments S1, S4 and S5, which have limited superior surface and minimal diaphragmatic contact. SARAY 1 (yellow) includes lateral left-lobe segments S2 and S3, with intermediate cranial orientation and partial diaphragmatic adjacency. SARAY 2 (red) comprises the posterior–superior right-lobe cluster S6–S8, representing the largest dome-facing surface and the greatest regional parenchymal extent within the grouping framework. This grouping was used to classify the dominant hepatic cyst for all analyses in the study

This hierarchical grouping was used as an exploratory framework for segment-level analysis in the present study (Fig. 1). It was designed to provide a structured anatomic representation of hepatic cyst location rather than a previously validated classification system. The term *SARAY* was selected as an acronym for “Segmental Anatomy–Related Y-Scale”, reflecting the superior–inferior (Y-axis) orientation underlying the classification. The geographic origin is acknowledged without methodological implication.

### Outcome Definition

The primary outcome was hepatopulmonary hydatidosis (HPH), defined as the presence of concomitant hepatic and pulmonary cystic echinococcosis detected at the time of initial diagnosis.

In this study, HPH was used as a clinical outcome label indicating baseline pulmonary involvement in patients with hepatic CE; it was not intended to establish a direct causal dissemination pathway from liver to lung.

Thoracic screening in patients with hepatic CE was based on chest radiography performed as part of routine clinical evaluation. Patients with suspected pulmonary hydatid involvement underwent thoracic CT for confirmation and characterization, and all patients classified as having pulmonary involvement (HPH/SPH) had thoracic CT confirmation. However, thoracic CT was not systematically performed in all hepatic-only cases; therefore, occult asymptomatic pulmonary involvement at baseline cannot be fully excluded in that group. No cases of newly detected pulmonary involvement were identified during follow-up. Pulmonary involvement required radiologic findings consistent with hydatid disease on thoracic imaging. Thoracic distribution (right vs left; upper vs lower lobes; unilateral vs bilateral) was recorded for secondary analysis.

### Data Collection

For each patient, demographic data, cyst characteristics (size, number, WHO stage, SARAY category), lobe/segment distribution, and multiorgan involvement were recorded. Pulmonary cyst characteristics (size and laterality) were extracted for patients with HPH or SPH.

**Table 1:** Baseline demographic and clinical characteristics

Variable	n (%) or Median (IQR)
Total patients	292 (100%)
Age (years)	46 (32–58)
Female sex	189 (64.7%)
Rural residence	125 (42.8%)
Follow-up duration (months)	65 (32–93)
Hepatic involvement (1)	244 (83.5%)
Hepatopulmonary (2)	23 (7.8%)
Single pulmonary (3)	18 (6.1%)
Other organ (4)	11 (3.7%)

**Table 2:** Hepatic cyst characteristics according to WHO stage and SARAY classification

Variable	n (%) or Median (IQR)
Hepatic cyst size (mm)	68 (50–90)
SARAY Class 0	41 (15.3%)
SARAY Class 1	31 (11.6%)
SARAY Class 2	195 (73%)
WHO Stage CE1–CE2 (active)	102 (38.2%)
WHO Stage CE3 (transitional)	54 (20.2%)
WHO Stage CE4–CE5 (inactive)	111 (41.5%)

**Table 3:** Distribution of WHO cyst stages in hepatic-only and hepatopulmonary disease

Group	Active (CE1–2) n (%)	Transitional (CE3) n (%)	Inactive (CE4–5) n (%)	*p*-value
Hepatic (1)	81 (33.2%)	52 (21.3%)	111 (45.5%)	0.0001
Hepatopulmonary (2)	21 (91.3%)	2 (8.7%)	0 (0.0%)

**Table 4:** Association between SARAY class and WHO cyst stage among hepatic cysts

SARAY class	CE1–2 (Active)	CE3 (Transitional)	CE4–5 (Inactive)	*p*-value
0	17.1%	31.7%	51.2%	
1	32.3%	16.1%	51.6%	
2	43.6%	18.5%	37.9%	0.017

**Table 5:** Multivariable logistic regression model for predictors of hepatopulmonary hydatidosis

Predictor	Category	β Coefficient	SE	*p*-value
Intercept	–	− 0.844	1.457	0.562
WHO Stage	1–3	− 2.178	0.647	0.001
SARAY	0–2	0.919	0.640	0.151

β coefficients are shown; adjusted odds ratios can be derived by exponentiation of β

### Statistical Analysis

Continuous variables were summarized as median and interquartile range (IQR) and compared using the Mann–Whitney U test. Normality was assessed using Shapiro–Wilk test. Categorical variables were compared using chi-square or Fisher’s exact tests. Statistical significance was defined as *p* < 0.05. All statistical analyses were conducted using SPSS version 27 (IBM Corp.)

### Predictive Model Development

The HepatoMAP model (Hepatic Predictive Model for Adjacency and Parasitic activity) was developed using binary logistic regression to evaluate predictors of HPH. To avoid incorporating patients without evaluable hepatic disease, analyses were restricted to the hepatic-only and HPH groups (n = 267). Candidate variables included WHO stage (grouped into active CE1–2, transitional CE3 and inactive CE4–5) and SARAY category (0–2). Calibration assessed using bootstrap-based calibration slope and intercept. Event-per-variable (EPV) considerations followed the recommendations of Peduzzi et al., ensuring minimal overfitting for the limited number of events [15]. WHO stage and SARAY category were prespecified based on biological and anatomical rationale; in multivariable analysis WHO stage remained statistically significant, whereas SARAY showed a positive but non-significant association. Nevertheless, SARAY was retained in the point-based score because of its structured univariable relationship with HPH, anatomical interpretability, and modest contribution to overall model discrimination. Point assignments for WHO stage and SARAY category, forming the HepatoMAP score (range 0–4), are detailed in Table 6.

**Table 6:** Point assignment for the HepatoMAP risk score

Variable	Points
WHO stage	CE1–2 = 2, CE3 = 1, CE4–5 = 0
SARAY class	0 = 0, 1 = 1, 2 = 2
Total Score Range	0–4
High-risk threshold	≥ 3 points

### Model Performance

Model discrimination was assessed using receiver operating characteristic (ROC) analysis. A simplified point-based score (HepatoMAP) was derived from regression coefficients and evaluated at prespecified thresholds. Sensitivity, specificity, and predictive values were calculated using exact binomial methods. Internal validation was performed using bootstrap resampling (1000 iterations), with optimism-corrected AUC values reported.

### Ethical Considerations

The study was approved by the institutional ethics committee (SAGETİK 2025-179), and the requirement for informed consent was waived owing to the retrospective design and anonymized data handling.

## Results

### Baseline and Hepatic Characteristics

A total of 292 patients were included. The median age was 46 years (IQR 32–58), and 64.7% were female. Rural residence accounted for 42.8% of the cohort. The median follow-up duration was 65 months (IQR 32–93).

Hepatic involvement was present in 83.5% of all cases (Table 1), while six patients had isolated extrahepatic disease (splenic, renal, or cerebral). Pulmonary involvement occurred in 10.3% of patients, of whom 7.8% had HPH detected at the time of initial diagnosis.

The median hepatic cyst size was 68 mm (IQR 50–90), and most cysts were located in SARAY 2 regions (segments 6–8), comprising 73.0% of all hepatic cysts (Table 2). According to the WHO-IWGE classification, 38.2% of cysts were *active* (CE1–2), *20.2% transitional (CE3)*, and *41.5% inactive (CE4–5)*.

A significant association was observed between SARAY category and WHO stage (χ2 = 12.08, *p* = 0.017). Active cysts occurred most frequently in SARAY 2, whereas inactive lesions were more commonly identified in SARAY 0 segments.

### Comparison Between Hepatic-Only and Hepatopulmonary Cases

Among the primary analytic cohort (n = 267), 23 patients (8.6%) had HPH at the time of initial diagnosis. For predictive modeling, analyses were restricted to patients with hepatic-only CE and HPH (n = 267), because patients with isolated pulmonary or non-hepatic extrahepatic disease lacked evaluable hepatic cyst characteristics.

Patients with HPH had a higher frequency of active cysts compared with hepatic-only patients (CE1–CE2: 91.3% vs 33.2%, *p* < 0.001; Table 3).

SARAY distribution also differed, with SARAY 2 cysts representing the majority of HPH cases (n = 21); however, this association did not remain statistically significant after adjusting for WHO stage (Table 4).

**Fig. 2:**
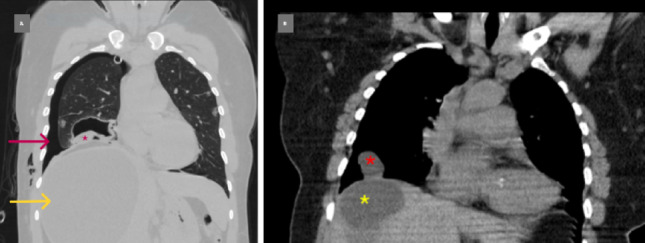
Representative computed tomography (CT) images of hepatopulmonary hydatidosis. **A** Coronal CT image showing a giant intact hydatid cyst in the hepatic dome (yellow arrow) and a ruptured hydatid cyst in the right lower lobe, with the detached germinative membrane visible (red asterisk). Associated right-sided pneumothorax is present (red arrow), and an apically positioned chest tube is present. **B** Coronal CT image demonstrating multiple hepatic hydatid cysts in the dome (yellow asterisk) and a hydatid cyst in the right lower lobe (red asterisk), located in close diaphragmatic proximity. This configuration illustrates the anatomic continuum between dome-adjacent hepatic segments and the ipsilateral basal lung region

Binary logistic regression identified WHO stage as the only independent predictor of HPH. Age, cyst size, residential setting (rural vs urban), and SARAY class showed no significant association with hepatopulmonary involvement at baseline. A modest association between SARAY and WHO stage was present (χ2 = 12.08, *p* = 0.017), indicating partial alignment between segmental topography and biological activity. Representative CT examples of dome-adjacent hepatic cysts and corresponding pulmonary involvement are shown in Fig. 2.

### Pulmonary Characteristics (HPH vs SPH)

Among patients with pulmonary CE, maximum cyst size differed significantly by group:

- Median 70 mm in isolated pulmonary disease (SPH)
- Median 50 mm in HPH (*p* = 0.047)

Pulmonary laterality (right-sided, left-sided, bilateral) and lobar distribution (upper vs lower lobes) showed no significant differences between SPH and HPH (all *p* > 0.05; Supplementary Table 1).

### Predictors of Hepatopulmonary Hydatidosis

In univariate analysis:

- Active WHO stage (CE1–2): OR 15.75
- SARAY-2 localization: OR 3.12

Increasing HPH frequency was associated with both cyst activity and SARAY category:

- SARAY-0: 0%
- SARAY-1: 6%
- SARAY-2: 22% among active cysts

In the multivariable logistic regression model, only WHO stage remained statistically significant (*p* = 0.001; OR 17.25, 95% CI 4.53–65.65), whereas SARAY showed a positive but non-significant trend (*p* = 0.151; OR 2.03, 95% CI 0.87–4.72) (Table 5). Given the limited number of HPH events, adjusted estimates should be interpreted with caution.

### HepatoMAP Model Performance

The combined HepatoMAP model (WHO stage + SARAY category) showed:

- AUC = 0.83 (95% CI 0.72–0.93)
- WHO-only AUC = 0.81
- SARAY-only AUC = 0.61 (See Fig. S1: ROC curves)

Bootstrap internal validation (1000 repetitions) demonstrated minimal performance degradation:

- Optimism-corrected AUC: 0.79
- Calibration slope: 0.97
- Intercept: 0.02
- Hosmer–Lemeshow *p *= 0.68

HL test should be interpreted cautiously given the low event count. Calibration results are shown in Fig. S2.

Decision curve analysis (Fig. S3) demonstrated net benefit across threshold probabilities between 10 and 20%, indicating that HepatoMAP is most informative when clinical suspicion for HPH lies within this range.

### HepatoMAP Scoring and Diagnostic Accuracy

The final point-based score ranged 0–4.

At the prespecified threshold ≥ 3, performance was:

- Sensitivity: 100%
- Specificity: 55.8%
- NPV: 100%
- PPV: 25%

Score distribution showed that no HPH case occurred at scores 0–1, whereas HPH frequency increased progressively at scores 2 (9.5%), 3 (23.5%), and 4 (25.7%). No HPH case scored < 3, supporting the use of ≥ 3 as the prespecified high-risk threshold and supporting its use as a rule-out tool rather than a diagnostic classifier. The distribution of HepatoMAP scores among hepatic-only and HPH cases is summarized in Table 7.

**Table 7:** Distribution of HepatoMAP risk scores among hepatic-only and hepatopulmonary cases

Score	HPH absent (n)	HPH present (n)
0	60	0
1	50	0
2	95	10
3	13	4
4	26	9

HPH, hepatopulmonary hydatidosis. Values represent absolute patient counts

## Discussion

In this retrospective cohort, we identified a structured relationship between hepatic cyst activity, segmental topography, and baseline hepatopulmonary involvement, which formed the basis of the HepatoMAP framework integrating parasite viability (WHO stage) with hepatic topology (SARAY). Prior studies of HPH have described dome involvement and right-lobe predominance, but none have systematically examined the risk of thoracic involvement at baseline at the level of individual hepatic segments [5, 6, 16]. The marked clustering of active (CE1–2) cysts in posterior–superior segments (S6–8) observed in our cohort provided the anatomical basis for SARAY and supported its exploratory integration with WHO stage into a unified predictive model.

The concentration of CE1–2 lesions in posterior–superior right-lobe segments is consistent with known hepatic volumetric asymmetries and with the possibility that larger posterior–superior territories may intercept a greater proportion of portal venous oncospheres, even though direct evidence of segment-level parasite deposition is lacking. These relationships should therefore be interpreted as biologically reasoned and hypothesis-generating, rather than as mechanistic proof.

Local mechanical factors may influence cyst evolution, but they are unlikely to be the sole explanation for the observed segmental pattern. Nevertheless, our data showed that SARAY 0–1 segments exhibited higher proportions of inactive (CE4–5) cysts, which could reflect earlier involution rather than intrinsic segmental predisposition.

Analogous spatial patterns in other hepatic conditions support the broader concept that hepatic disease foci do not distribute randomly. Hepatocellular carcinoma and colorectal liver metastases most frequently involve the right hepatic lobe, reflecting volumetric and inflow asymmetries rather than stochastic spread [17, 18]. These patterns do not imply shared biology with echinococcosis, but illustrate that hepatic diseases often distribute non-randomly across segments.

Although posterior–superior segments may represent a region of greater viable cyst burden, location alone cannot explain all patterns of thoracic involvement. Classical surgical series strongly emphasized the mechanical relationship between dome-adjacent hepatic cysts and the diaphragm [5, 16, 19]. However, the heterogeneous laterality and lobar distribution of pulmonary cysts observed in our cohort argues against a purely transdiaphragmatic process and supports the likelihood of mixed pathways (Supplementary Table 1). Although direct mechanistic data in humans are limited, the combination of diffuse pulmonary distribution, involvement of non-diaphragmatic lung regions, and high prevalence of CE1–2 among HPH cases strongly suggests that systemic venous spread operates alongside local mechanical penetration.

The integration of cyst viability and segment-level characteristics into a unified predictive framework may have several potential clinical implications. The absence of HPH among patients with low HepatoMAP scores suggests that routine thoracic imaging may be deferred in selected low-risk cases, although this observation requires external validation.

The observation that virtually all HPH cases in our cohort involved active (CE1–2) cysts, and that these were predominantly located within posterior–superior segments, underscores the importance of carefully assessing these regions in patients with newly diagnosed hepatic echinococcosis. Dome-adjacent, biologically active cysts may warrant heightened attention because they combine two risk-enhancing features: a greater viable cyst burden and the potential for local diaphragmatic penetration. Early recognition of baseline pulmonary involvement in such patients could meaningfully influence operative planning—particularly decisions regarding combined thoracic–abdominal approaches, anesthetic precautions, and the timing of antiparasitic therapy. Case reports have described intraoperative rupture of pulmonary or hepatic hydatid cysts during positive-pressure ventilation, leading to airway obstruction, pleural contamination, and even fatal anaphylaxis [7, 20, 21]. Although these observations support a cautious approach to perioperative imaging and anesthetic planning, controlled data are lacking. However, evidence linking preoperative albendazole to increased rupture risk remains inconsistent, and most data derive from small retrospective series.

The segmental patterns observed here raise questions about whether cyst location may influence the natural history of disease or responsiveness to medical therapy. In our cohort, SARAY 0–1 regions exhibited a higher proportion of inactive (CE4–5) cysts at the time of diagnosis, a pattern that may reflect earlier involution rather than intrinsic segmental predisposition. No published CE study has directly compared involution rates across hepatic segments, so this interpretation is hypothesis-generating rather than evidence-based.

Previous surgical and imaging series have generally described the hepatic distribution of cystic echinococcosis at a coarse topographic level rather than by individual segments. Kayal and Hussain reported that the vast majority of hepatic hydatid cysts in their prospective series involved the right lobe, without further segmental stratification [22]. Kendyala and Narayanan similarly highlighted right-lobe and dome predominance in a recent pictorial review, but localization remained lobar or regional rather than segment-based [23]. In a large single-center cohort, Azizoğlu and colleagues summarized multiple studies in which 45–85% of liver cysts were located in the right lobe, again using right/left or bilateral categories instead of Couinaud segments [24]. For dome-adjacent disease, Abdollahi et al. classified hepatic lesions as “liver dome or segments 7–8” when comparing thoracotomy versus laparotomy, effectively treating the posterior–superior surface as a single surgical zone [25]. While these approaches consistently support right-lobe and dome predominance, they implicitly assume that all lesions within a given lobe or dome share similar biological behavior and dissemination risk.

In contrast, the SARAY framework used in the present study formalizes segment-level topology into three ordered groups (S1/4/5, S2/3, S6–8), allowing us to demonstrate that posterior–superior segments (SARAY 2) harbor a disproportionately high burden of active (CE1–2) cysts, whereas medial segments (SARAY 0) accumulate more inactive (CE4–5) lesions. This segment-based resolution reveals intra–right-lobe heterogeneity that is not captured by lobar or dome/non-dome classifications and provides a structured anatomical basis for exploratory risk modeling in HepatoMAP.

Importantly, adopting a segment-based framework may provide a more reproducible measure of hepatic localization than traditional right/left or dome/non-dome classifications, enabling more consistent anatomical comparisons across future studies.

Finally, these findings highlight the potential for personalized risk stratification in cystic echinococcosis. Current management guidelines rely primarily on WHO stage and cyst size, which remain essential determinants of therapy. However, our results suggest that integrating segmental information—a dimension rarely considered—may refine risk assessment for HPH when interpreted alongside cyst activity. Whether such refinements improve patient outcomes, reduce unnecessary imaging, or optimize the timing of therapeutic interventions will require prospective validation.

Beyond risk stratification, determining which patients with hepatic CE warrant thoracic imaging carries substantial therapeutic relevance, as management pathways differ markedly between isolated hepatic disease and combined hepatopulmonary involvement. However, when pulmonary hydatid disease coexists, preoperative benzimidazole therapy remains controversial. Several reports have suggested that albendazole may weaken the cyst wall or alter pericystic tissue, potentially increasing the risk of rupture and making thoracic surgery more challenging [8, 26, 27]. Although this association remains debated and is not supported by controlled trials, undetected pulmonary cysts also pose intraoperative risks: positive-pressure ventilation can facilitate cyst rupture, leading to pleural contamination, empyema, bronchobiliary communication, or anaphylactic reactions, all of which have been repeatedly reported in surgical case series [28].

Our data further emphasize this point. Because HPH in our cohort was overwhelmingly associated with active (CE1–2) hepatic cysts, and because thoracic involvement was frequently asymptomatic at presentation, missing pulmonary disease at the time of hepatic CE diagnosis carries meaningful clinical consequences. Early detection allows coordination of surgical timing, avoidance of prolonged preoperative albendazole exposure in patients who may require thoracic intervention, and prevention of intraoperative complications related to occult cyst rupture. These considerations underscore the value of a hepatic-stage triage tool for identifying patients who warrant thoracic imaging at baseline. Its high sensitivity and negative predictive value in this cohort suggest that patients with inactive or favorably located cysts (low HepatoMAP scores) may not require routine thoracic CT, whereas those with active cysts in posterior–superior segments (WHO CE1–2 combined with SARAY-2) represent a subgroup at substantially higher risk. Because predictive values are prevalence-dependent, these findings must be interpreted cautiously and validated in external cohorts. Nevertheless, integrating segmental anatomy with parasite viability may provide a structured and clinically intuitive basis for imaging decisions.

A reciprocal observation also emerges. In our cohort, 54.7% of patients with pulmonary CE had concomitant hepatic cysts, a proportion slightly higher than the 30–40% range reported in prior series. This finding supports routine abdominal evaluation in all newly diagnosed pulmonary CE cases. Because hepatic cysts in this setting predominantly involve the right lobe, ultrasonography—highly sensitive for dome-adjacent and subcapsular lesions—serves as an effective first-line modality, reserving CT or MRI for left-lobe lesions, equivocal findings, or preoperative staging.

### Strengths & Limitations

Several features strengthen the interpretability of our findings. This study applies segment-level analysis to hepatic cystic echinococcosis, moving beyond traditional lobar or dome-based descriptions. This approach allowed us to examine structured spatial patterns that would have been less apparent under conventional classifications.

A second strength is the use of a single institutional radiology workflow for case identification and baseline imaging assessment, which reduced variation in segment assignment and WHO staging, although inter-reader variability cannot be fully excluded. The predictive model used prespecified, clinically interpretable variables, limiting unnecessary model complexity and supporting practical applicability.

Several limitations warrant consideration. The retrospective, single-center design limits generalizability and precludes causal inference. Physiologic interpretations remain indirect. The modest number of HPH cases limited statistical power and precluded more complex modeling. CE3a and CE3b lesions were combined into a single transitional group (CE3) for analysis; potential differences in behavior between these subclasses could not be assessed. We analyzed only the dominant hepatic cyst per patient (most active or largest), which may overlook contributions from smaller or anatomically discordant concomitant lesions and introduce within-patient selection bias.

An important additional limitation relates to thoracic imaging practices. Thoracic screening in patients with hepatic CE was based on routine chest radiography, and thoracic CT was performed selectively in patients with suspected pulmonary involvement. All patients classified as having pulmonary involvement (HPH/SPH) had thoracic CT confirmation; however, thoracic CT was not systematically performed in all hepatic-only cases at baseline, particularly among those managed medically. Accordingly, subclinical or asymptomatic pulmonary involvement may have been missed in a subset of hepatic-only patients. This limitation introduces the possibility of outcome misclassification and likely biases the observed associations toward the null rather than exaggerating risk estimates. Notably, no patient developed newly detected pulmonary CE during follow-up, suggesting that missed baseline disease—rather than delayed dissemination—is the more plausible concern.

Predictive performance is prevalence-dependent, and the apparent rule-out characteristics of HepatoMAP should be interpreted in that context. External validation in independent cohorts with standardized thoracic imaging protocols is required before clinical implementation.

### Future Directions

Future research should prioritize prospective, multicenter validation of HepatoMAP, ideally in cohorts with standardized imaging protocols and sufficient numbers of hepatopulmonary cases to allow robust multivariable modeling. Such studies would help clarify whether the incremental value of SARAY observed here persists across varying disease prevalence and radiologic practices, and whether segment-based predictors remain stable in populations with different endemic exposures or treatment patterns. External validation would also enable calibration analysis—an essential step before clinical implementation—and permit exploration of optimized decision thresholds tailored to specific healthcare settings.

A second avenue for investigation concerns the biological underpinnings of segment-level differences in cyst involution. In our cohort, SARAY 0–1 regions contained a disproportionately high number of inactive (CE4–5) cysts. This observation does not imply that certain segments predispose inactive disease; rather, it raises the possibility that local factors—such as lower overall parasite load in smaller segments, reduced diaphragmatic shear, or microenvironmental conditions favoring earlier pericyst stabilization—may facilitate spontaneous regression. These mechanisms remain unproven, but they generate a clinically meaningful hypothesis: whether medical therapy could be favored or prolonged in selected patients with SARAY 0–1 cysts, provided that standard WHO-based indications are met and no surgical criteria are present. Prospective longitudinal imaging studies comparing segment-specific involution rates under benzimidazole therapy will be essential before any segment-informed modification to treatment algorithms can be recommended.

Future work should also examine whether biological markers of cyst activity—including circulating antigens, cytokine signatures, or advanced imaging-derived surrogates such as diffusion metrics—can augment segmental anatomy to refine risk prediction. Integrating such biomarkers with HepatoMAP may improve early identification of patients at increased risk for baseline pulmonary involvement and reduce reliance on low-yield imaging.

Finally, the reciprocal observation that more than half of patients with pulmonary CE in our cohort had concomitant hepatic cysts supports evaluation of bidirectional staging strategies. Prospective studies comparing standardized abdominal and thoracic imaging algorithms for newly diagnosed CE—both hepatic and pulmonary—may help determine the most efficient and cost-effective approach to comprehensive disease staging. Ultimately, combining segment-based anatomy, cyst viability, and systemic screening principles could form the foundation of a precision-staging framework in echinococcosis, aligning diagnostic intensity with individualized risk.

## Conclusion

In this cohort of patients with hepatic cystic echinococcosis, baseline hepatopulmonary hydatidosis was strongly associated with cyst biological activity and showed a structured, but non-independent, relationship with segmental topography. The HepatoMAP model—combining parasite viability (WHO stage) with segment-level hepatic topology (SARAY)—provides a simple and clinically interpretable framework for estimating baseline HPH risk in this dataset. Its high negative predictive value suggests that HepatoMAP may help reduce unnecessary thoracic imaging, although this finding should be interpreted cautiously given the retrospective design, modest event count, and incomplete baseline thoracic CT assessment in hepatic-only cases. Prospective multicenter external validation is required before routine clinical implementation.

## Supplementary Information

Below is the link to the electronic supplementary material.

Supplementary Material 1

## Data Availability

The datasets generated and/or analyzed during the current study are available from the corresponding author on reasonable request.

## References

[1] Ammann R, Eckert J (1995) Echinococcus and hydatid disease

[2] Brunetti E, Kern P, Vuitton DA (2010) Expert consensus for the diagnosis and treatment of cystic and alveolar echinococcosis in humans. Acta Trop 114(1):1–16. 10.1016/j.actatropica.2009.11.001 19931502 10.1016/j.actatropica.2009.11.001

[3] Stojković M, Weber TF, Junghanss T (2018) Clinical management of cystic echinococcosis: state of the art and perspectives. Curr Opin Infect Dis 31(5):383–392. 10.1097/QCO.0000000000000485 30124496 10.1097/QCO.0000000000000485

[4] Nunnari G (2012) Hepatic echinococcosis: clinical and therapeutic aspects. World J Gastroenterol 18(13):1448–1458. 10.3748/WJG.V18.I13.1448 22509076 10.3748/wjg.v18.i13.1448 PMC3319940

[5] Kilani T et al (2001) Hydatid disease of the liver with thoracic involvement. World J Surg 25(1):40–45. 10.1007/S002680020006 11213155 10.1007/s002680020006

[6] Aribas OK, Kanat F, Turk E, Kalayci MU (2002) Comparison between pulmonary and hepatopulmonary hydatidosis. Eur J Cardio-thoracic Surg 21(3):489–496. 10.1016/S1010-7940(01)01140-X 10.1016/s1010-7940(01)01140-x 11888769

[7] Lupia T et al (2021) Pulmonary echinococcosis or lung hydatidosis: a narrative review. Surg Infect 22(5):485–495. 10.1089/SUR.2020.197 10.1089/sur.2020.197 33297827

[8] Rawat S, Kumar R, Raja J, Singh R, Thingnam SS (2019) Pulmonary hydatid cyst: review of literature. J Family Med Prim Care 8(9):2774. 10.4103/JFMPC.JFMPC_624_19 31681642 10.4103/jfmpc.jfmpc_624_19 PMC6820383

[9] Sayek I, Onat D (2001) Diagnosis and treatment of uncomplicated hydatid cyst of the liver. World J Surg 25(1):21–27. 10.1007/S002680020004 11213152 10.1007/s002680020004

[10] Pedrosa I, Saíz A, Arrazola J, Ferreirós J, Pedrosa CS (2000) Hydatid disease: radiologic and pathologic features and complications. Radiographics 20(3):795–817. 10.1148/RADIOGRAPHICS.20.3.G00MA06795 10835129 10.1148/radiographics.20.3.g00ma06795

[11] Fasel JHD, Gailloud P, Terrier F, Mentha G, Sprumont P (1996) Segmental anatomy of the liver: a review and a proposal for an international working nemenclature. Eur Radiol 6(6):834–837. 10.1007/BF00240684 8972319 10.1007/BF00240684

[12] Lim MC, Tan CH, Cai J, Zheng J, Kow AWC (2014) CT volumetry of the liver: where does it stand in clinical practice? Clin Radiol 69(9):887–895. 10.1016/j.crad.2013.12.021 24824973 10.1016/j.crad.2013.12.021

[13] Altintas N (2003) Past to present: echinococcosis in Turkey. Acta Trop 85(2):105–112. 10.1016/S0001-706X(02)00213-9 12606087 10.1016/s0001-706x(02)00213-9

[14] Ok ÜZ, Kilimcioğlu AA, Özkol M (2020) Cystic echinococcosis in humans in Turkey. Mikrobiyol Bul 54(3):510–522. 10.5578/MB.69712 32755525 10.5578/mb.69712

[15] Peduzzi P, Concato J, Kemper E, Holford TR, Feinstem AR (1996) A simulation study of the number of events per variable in logistic regression analysis. J Clin Epidemiol 49(12):1373–1379. 10.1016/S0895-4356(96)00236-3 8970487 10.1016/s0895-4356(96)00236-3

[16] Manterola C, Otzen T (2017) Hepatic echinococcosis with thoracic involvement. Clinical characteristics of a prospective series of cases. Ann Hepatol 16(4):599–606. 10.5604/01.3001.0010.0305 28611263 10.5604/01.3001.0010.0305

[17] Renzulli M et al (2022) Segmental distribution of hepatocellular carcinoma in cirrhotic livers. Diagnostics. 10.3390/DIAGNOSTICS12040834 35453882 10.3390/diagnostics12040834 PMC9032124

[18] Savvakis S et al (2024) Streamline flow of the portal vein affects the distribution of colorectal cancer metastases: clinical reality or just a belief? A systematic review and meta-analysis. Cancers 16(23):3902. 10.3390/CANCERS16233902 39682091 10.3390/cancers16233902 PMC11639830

[19] Aribaş BK, Dingil G, Köroǧlu M, Üngül Ü, Zarali AC (2011) Liver hydatid cyst with transdiaphragmatic rupture and lung hydatid cyst ruptured into bronchi and pleural space. Cardiovasc Intervent Radiol. 10.1007/S00270-009-9734-0 19847481 10.1007/s00270-009-9734-0

[20] Gupta R, Wadhawan S, Bhadoria P (2013) Intraoperative endobronchial rupture of pulmonary hydatid cyst: an airway catastrophe. J Anaesthesiol Clin Pharmacol 29(1):111. 10.4103/0970-9185.105817 23493935 10.4103/0970-9185.105817 PMC3590514

[21] Anthi A, Katsenos C, Georgopoulou S, Mandragos K (2004) Massive rupture of a hepatic hydatid cyst associated with mechanical ventilation. Anesth Analg 98(3):796–797. 10.1213/01.ANE.0000099722.80132.17 14980939 10.1213/01.ane.0000099722.80132.17

[22] Kayal A, Hussain A (2014) A comprehensive prospective clinical study of hydatid disease. ISRN Gastroenterol 2014:1–5. 10.1155/2014/514757 10.1155/2014/514757 PMC3966475 24734188

[23] Kendyala S, Narayanan R (2024) Encysted odyssey: a clinical and pictorial analysis of hydatid cysts from head to toe. Cureus. 10.7759/CUREUS.61180 38933644 10.7759/cureus.61180 PMC11205266

[24] Azizoğlu M et al (2024) Analysis of complications of a neglected disease: 13 years of experience with liver hydatid cysts in a high-volume hospital. Medicina 60(10):1696. 10.3390/MEDICINA60101696 39459483 10.3390/medicina60101696 PMC11509507

[25] Abdollahi M, Karkeabadi N, Pourahmadi Y, Rezaee V, Aghajanzadeh M. Liver dome hydatid cyst management: laparotomy or thoracotomy. 13(1)

[26] Kurkcuoglu IC, Eroglu A, Karaoglanoglu N, Polat P (2002) Complications of albendazole treatment in hydatid disease of lung. Eur J Cardiothorac Surg 22(4):649–650. 10.1016/S1010-7940(02)00396-2 12297195 10.1016/s1010-7940(02)00396-2

[27] Dehkordi AB et al (2019) Albendazole and treatment of hydatid cyst: Review of the literature. Infect Dis Drug Targets. 10.2174/1871526518666180629134511 10.2174/1871526518666180629134511 29956639

[28] Ferreres AR (2020) Management of thoracic hydatid disease and its complications. Surg Manag Parasit Dis. 10.1007/978-3-030-47948-0_10

