# Supplementary material for: Predictors of Concomitant Pulmonary Involvement in Hepatic Cystic Echinococcosis: A Clinical Risk Stratification Model
Source: Acta Parasitol. 2026 May 2;71(3):98. doi: 10.1007/s11686-026-01291-4 (PMC13135539; doi:10.1007/s11686-026-01291-4)
Supplement: Supplementary file 1 — Supplementary Material 1 [file 11686_2026_1291_MOESM1_ESM.docx]

**Supplementary Table 1:** **Radiologic characteristics of pulmonary cysts in hepatopulmonary (HPH) versus single pulmonary hydatid disease (SPH)**

| Parameter | HPH (n = 23) | SPH (n = 18) | p-value |
| --- | --- | --- | --- |
| Pulmonary cyst size (mm), Median (IQR) | 50 (40–60) | 70 (42–90) | 0.047 (Mann–Whitney U) |
| Pulmonary side (Right / Left / Bilateral) | 11 / 9 / 3 | 6 / 9 / 3 | 0.234 (Chi-square) |
| RLL involvement, n (%) | 13 (57%) | 5 (28%) | 0.106 (Fisher’s exact) |
| Lower-lobe involvement (RLL + LLL), n (%) | 18 (78%) | 10 (56%) | 0.196 (Fisher’s exact) |


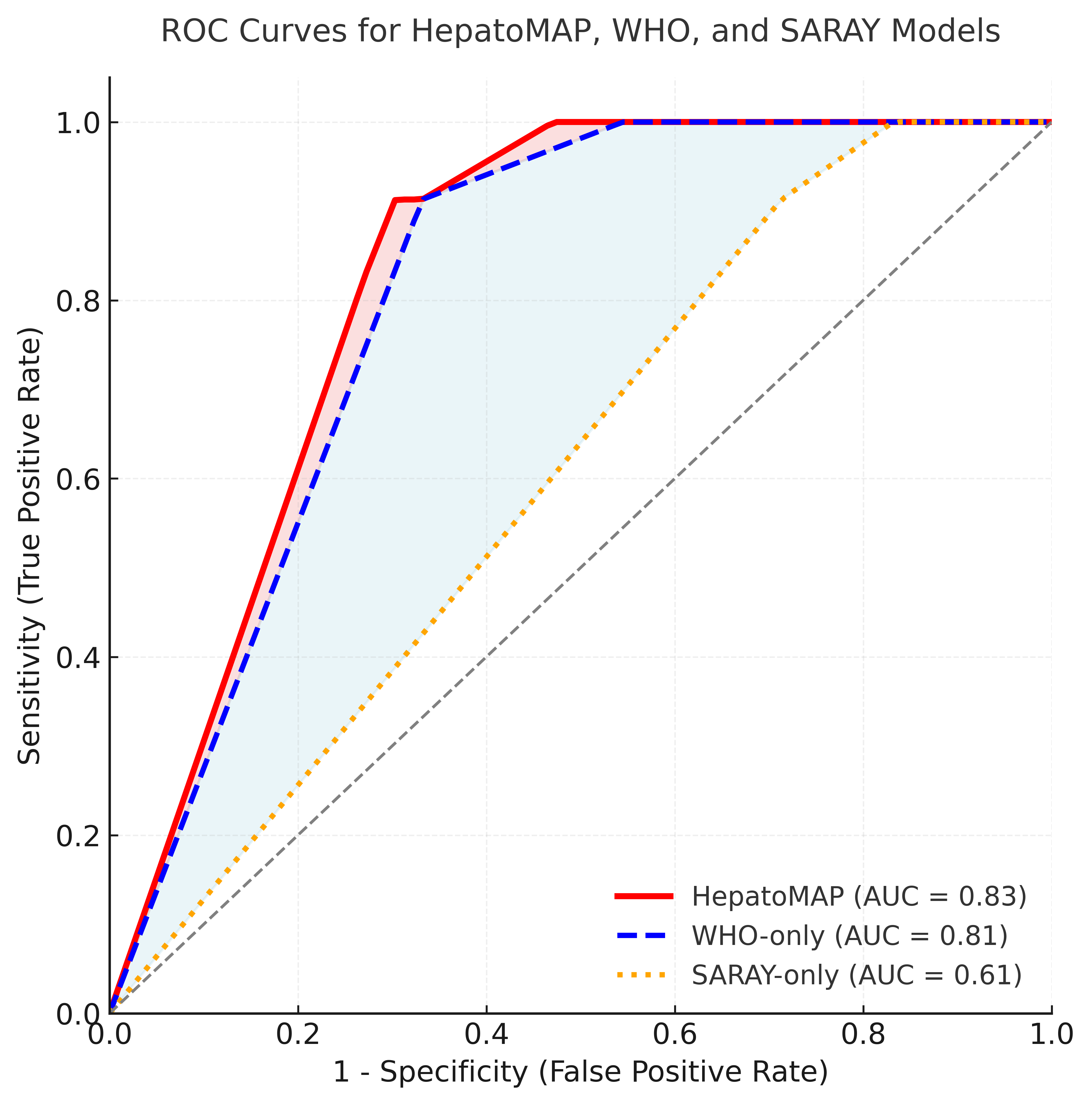


**Figure S1. Receiver Operating Characteristic (ROC) Curves for HepatoMAP, WHO-Only, and SARAY-Only Models**

ROC curves depict the discriminative performance of the combined HepatoMAP model (area under the curve [AUC] = 0.83), the WHO stage–only model (AUC = 0.81), and the SARAY-only model (AUC = 0.61) for predicting hepatopulmonary hydatidosis.


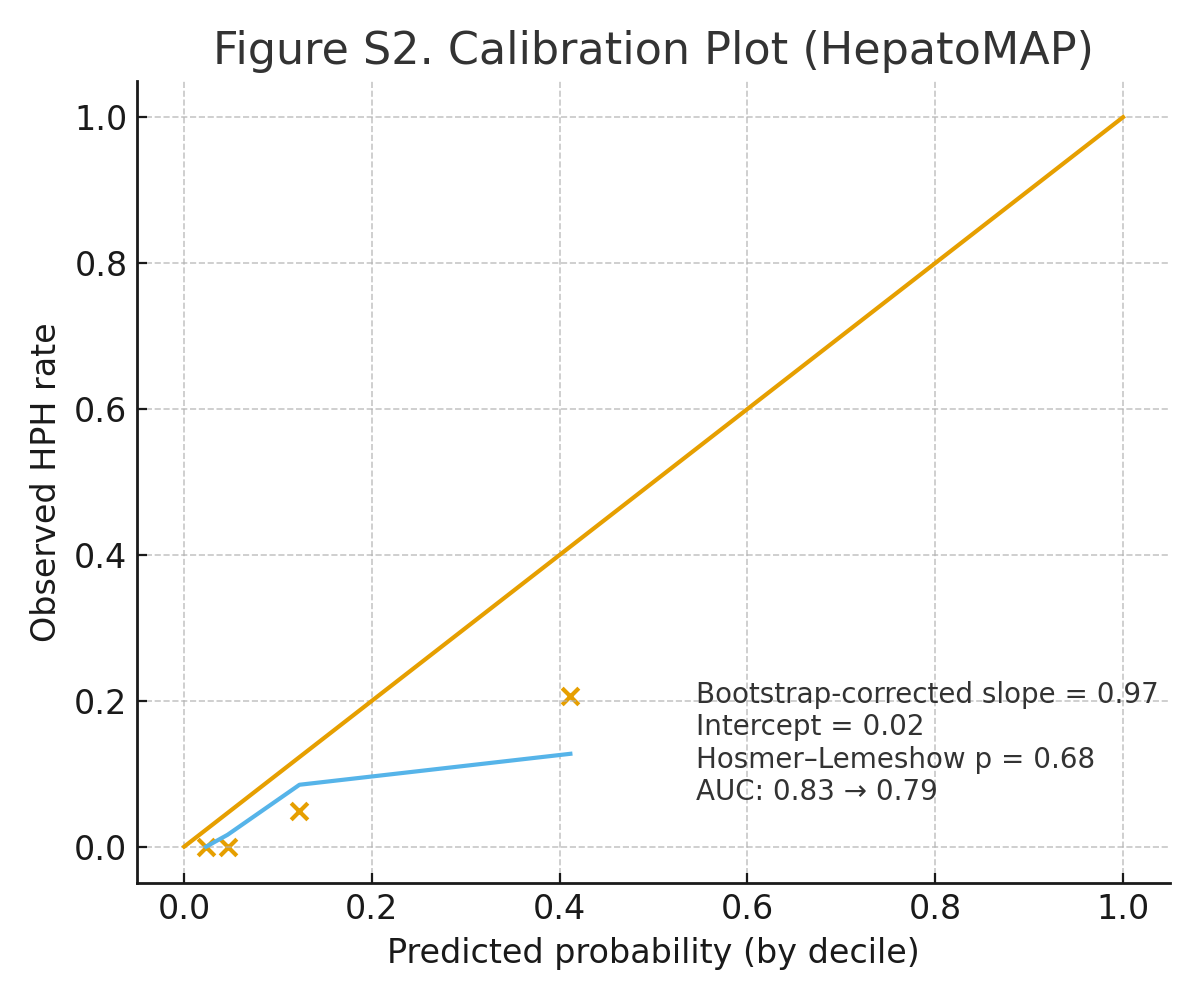


**Figure S2. Calibration Plot for the HepatoMAP Model**

Calibration plot showing agreement between predicted probabilities and observed outcomes across deciles of risk. The bootstrap-corrected calibration slope was 0.97 with an intercept of 0.02. The optimism-corrected AUC was 0.79, and the Hosmer–Lemeshow goodness-of-fit test yielded p = 0.68.


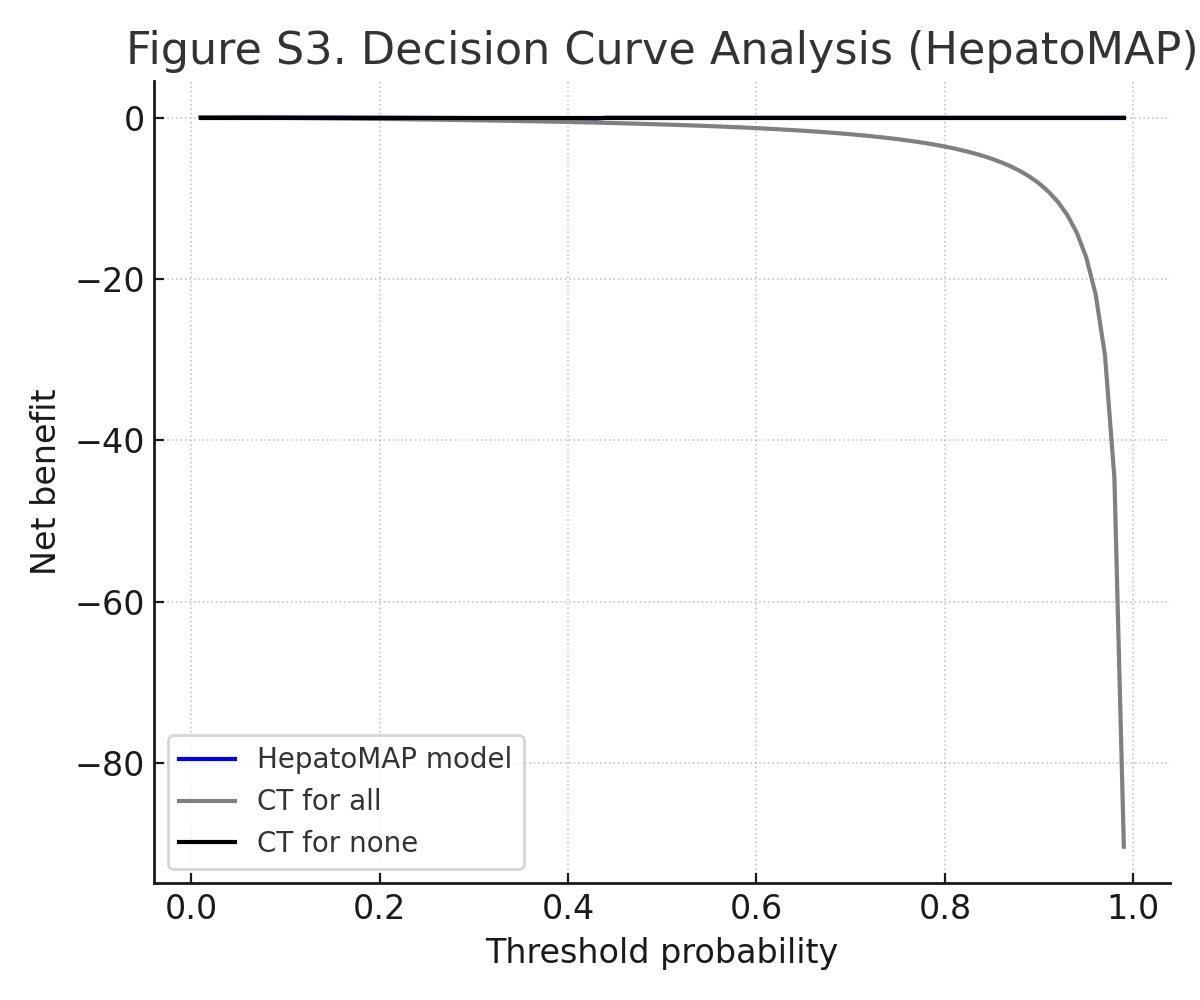


**Figure S3. Decision Curve Analysis for the HepatoMAP Model**

Decision curve analysis illustrating net benefit across a range of threshold probabilities for the HepatoMAP model compared with “CT for all” and “CT for none” strategies.
